# Prediction of severity and outcomes of colon ischaemia using a novel prognostic model: a clinical multicenter study

**DOI:** 10.1080/07853890.2021.1990391

**Published:** 2021-11-02

**Authors:** Xinbo Ai, Yuping Chen, Jiajian Qian, Bin Zhou, Zhenjiang Wang, Yanjun Zhang, Aimin Li, Feiyue Gong, Wensheng Pan, Bo Shen, Side Liu

**Affiliations:** a Department of Gastroenterology, Nanfang Hospital, Southern Medical University, Guangzhou, China; b Department of Gastroenterology, Zhuhai People’s Hospital (Zhuhai Hospital Affiliated with Jinan University), Zhuhai, China; c Department of Pharmacy, Zhuhai People’s Hospital (Zhuhai Hospital Affiliated with Jinan University), Zhuhai, China; d Department of Pathology, Zhuhai People’s Hospital (Zhuhai Hospital Affiliated with Jinan University), Zhuhai, China; e Department of Intensive Care Unit, Zhuhai People’s Hospital (Zhuhai Hospital Affiliated with Jinan University), Zhuhai, China; f Department of Gastroenterology, Zhejiang Provincial Hospital, People’s Hospital of Hangzhou Medical College, Hangzhou, China; g The Inflammatory Bowel Disease Center at Columbia, Columbia University Irving Medical Center, New York, NY, USA

**Keywords:** Colon ischaemia, Oakland score, colonoscopy, histopathology, surgery, prognostic model

## Abstract

**Objective:**

To identify risk factors of disease severity and between mild and severe colon ischaemia (CI) patients and to improve clinical outcomes, this study aimed to explore a novel scoring model.

**Methods:**

Retrospective analyses of hospital records between January 2009 and December 2019 were included. Clinical manifestations, mortality, Oakland score, laboratory tests, colonoscopy, and histopathology were collected. Risk factors of severe CI were determined by univariate and multivariate logistic regression and used for the predicting model.

**Results:**

A total of 203 patients with CI were included. Serum C-reactive protein (CRP) and albumin ratio (CAR) were much higher in the severe CI group compared with that of the mild CI group (3.33 ± 1.78 versus 0.68 ± 0.97, *p* < .001). The Oakland score was much higher in the severe CI group (12.00 ± 3.02 versus 8.77 ± 1.63, *p* < .001). The histopathological finding of fibrin thrombi was an independent risk factor that predicted poor outcomes (20.00% versus. 1.09%, *p* < .001). Patients present with CAR ≥3.33, Oakland score ≥12, and histopathological fibrin thrombi were independent risk factors. In addition, the final scoring model was 0.042 × Oakland score + 1.040 × CAR + 3.412 × fibrin thrombi, the area under the curve (AUC) was 0.960 (95% confidence interval:0.930–0.990), and the sensitivity and specificity of the novel scoring model were 95% and 92%, respectively.

**Conclusions:**

The novel prognostic model was established to predict CI severity and clinical outcomes efficiently.

**Key messages**
In this article, we discuss the scoring model for clinical outcomes of colon ischaemia patients.
In our study, the sensitivity and specificity of a novel scoring model are very high.
Thus, laboratory tests (CRP albumin ratio), Oakland score, and histopathological findings (fibrin thrombi) can be assessed efficiently for colon ischaemia outcomes.

## Introduction

CI is a vascular disease of mesenteric ischaemia, loss of blood supply, and ischaemic injuries, manifested as acute or chronic onset. CI is a quite common sign of gastrointestinal injuries; moreover, it is the second-most common cause of acute lower gastrointestinal bleeding (LGIB) [1]. CI is a spectrum of diseases, ranging from transient colitis (50–60%) to gangrene (10–15%) or fulminate universal colitis (<5%) [2]. Transient colitis on endoscopy usually has a favourable outcome. However, the general inpatient mortality is 11.5%, and 17.0% of patients with CI require surgery [3].

There is no consensus on whether biopsies should be routinely performed. The American College of Gastroenterology (ACG) guidelines state that biopsies are not helpful and could entail a high risk of perforation. However, colonoscopy is strongly recommended by most gastroenterologists, and it is safe when performed by experienced endoscopists [4]. In a retrospective study of 659 CI cases, there was no reported case of perforation secondary to colonoscopy [5]. On the other hand, histopathological evaluation is essential for diagnosis and differential diagnosis of CI in China, due to the frequency of various infectious and inflammatory aetiologies.

Regrettably, there are limited clinical studies of a prognostic tool for CI patients. CRP is a positive reactant synthesized by the liver during the acute phase and increases in level in response to inflammation and infection [6]. In our current clinical practice, CRP is widely used to assess intestinal inflammatory activity. However, CRP is not so specific for the evaluation of intestinal damage [7]. For this purpose, we intend to determine a novel serum marker for clinical use.

Serum albumin can maintain colloid pressure and transport free fatty acids, bilirubin, and drug metabolites [8]. Serum albumin may decrease significantly due to acute inflammation, but nutritional status influences albumin level. Low albumin level is mostly associated with chronic disease, frequently correlated with nutritional status [9]. CRP and albumin ratio (CAR), as a new inflammation-based prognostic score, has been demonstrated to show prognostic value in hepatocellular carcinoma (HCC), oesophageal squamous-cell carcinoma, and small-cell lung cancer [10,11]. Little literature is available on CAR for clinical prediction and outcomes from CI.

The Oakland score is a risk assessment score of acute LGIB and can be widely used to evaluate bleeding whether massive or minor [12]. Patients with a score >8 points are classified as having massive bleeding, and those patients were more likely to be hospitalized or to have much higher risk of severe complications or even death [13]. Few studies have been done on Oakland score and its correlated relationship with disease pattern and clinical outcome in CI patients.

Little attention has been devoted to a predictive tool to assess disease severity and clinical outcome, so we hypothesized that certain demographic and clinical factors may predispose the patients to a negative prognosis. In this paper, a new prognostic tool will be developed efficiently for CI patients.

## Patients and methods

### Ethics

This study was reviewed and approved by the Institutional Review Board (IRB) of Nanfang Hospital, Southern Medical University (Ethics Review Code: NFEC-201609-K6). Hospital Information Software (HIS) was used to collect clinical documentations. Personal information was made anonymous before analysis.

### Patients and study design

Two hundred and three patients with CI were enrolled in our retrospective study from January 2009 to December 2019 at Nanfang Hospital, Southern Medical University, Zhuhai People’s Hospital (Zhuhai Hospital affiliated with Jinan University), and Zhejiang Provincial Hospital. CI diagnosis was based on the presence of clinical manifestations and endoscopic and pathological findings [14].

### Inclusion and exclusion criteria

**Figure 1.**
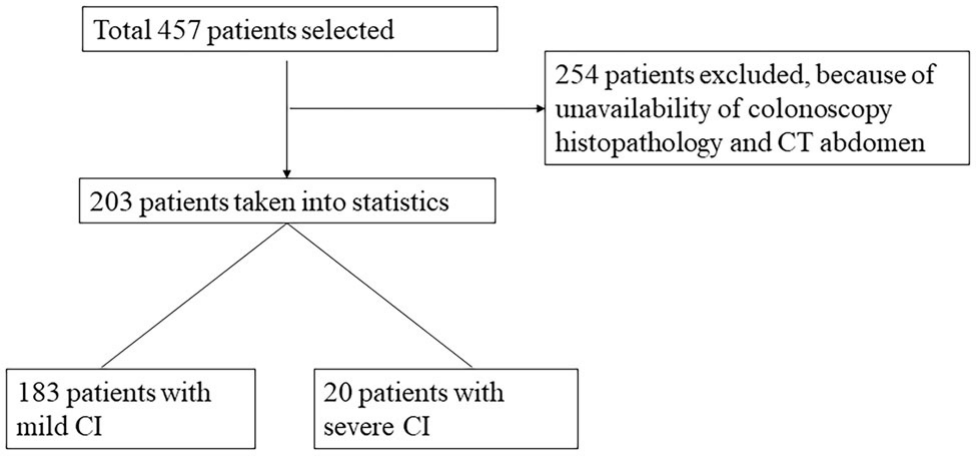
Flowchart of patients in development cohort.

Inclusion criteria included (1) a colonoscopic or surgical evaluation of the bowel; (2) a biopsy or surgical pathology finding that consistent with the diagnosis of CI and (3) a CT abdomen of bowel evaluation for CI. All 203 patients submitted a stool culture, patients with bacterial colitis and inflammatory bowel disease were excluded (Figure 1). Written informed consent was obtained before endoscopic examinations. All patients with CI received either urgent or elective colonoscopy with endoscopic biopsies (Olympus CF-240I, CF-H260AI, Tokyo, Japan; Fujinon EC-580RD, EC-590WM, Tokyo, Japan).

All patients were divided into mild and severe CI groups. The mild group included patients who did not require surgery and could achieve positive outcomes. The severe group included patients who required abdominal surgeries or died.

Gender, age, underlying diseases, clinical manifestations (temperature >38.0 °C), CAR, Oakland score, colonoscopy and histopathological findings were all collected into our medical documentations.

Medical treatment included empirical antibiotics, nutritional supply, fluid therapy, and restrictive blood transfusion if necessary.

Surgery was indicated for patients with severe peritonitis, intestinal perforation, massive bleeding, or clinical deterioration.

### Diagnosis

We defined urgent colonoscopy as colonoscopy required within 24 h and standard colonoscopy as not required within 24 h. The category of colonoscopy biopsy findings was divided into mucosal erosion, ulceration, haemorrhage, necrosis, inflammatory cells infiltration, dilated vessel, thickening vessels, and fibrin thrombi.

### Colonoscopy findings

Colon ischaemia was divided into two types, according to endoscopic findings: (1) a “non-ulcer” type for findings of pale, friable, or oedematous mucosa with petechial haemorrhages, scattered erosion, or segmental erythema; (2) an “ulcer” type for findings of blue-black mucosal nodules, or mucosal granularity with deep ulcerations [15].

### Histopathology

All tissue samples obtained from these 203 patients with forceps or surgical specimens were fixed in 4% buffered paraformaldehyde, processed routinely, embedded in paraffin, and stained with hematoxylin–eosin (HE) staining. Two senior pathologists were blinded to examine the slides under light microscopy and recorded the histopathological report (Olympus, Tokyo, Japan).

### Definition of mild and severe colitis

The diagnosis of CI was made based on clinical manifestations and negative stool studies for infections, CT scanning, colonoscopy with histopathological findings. Patients were divided in two groups. The mild group included patients who could achieve positive outcomes with medical treatment. The severe group included patients who required abdominal surgeries or died.

### Statistical analysis

Descriptive variables were expressed as means with standard deviation and were compared using Student’s *T* test. Categorical variables were expressed as numbers and proportion. The χ^2^ statistics were used to compare most categorical numbers, and Fisher’s exact test was used for small numbers. Multiple logistic regression analysis was used to determine risk factors of CI. The area under the receiver operating characteristics (ROC) curve (AUC) represented discrimination of the model. Confidence intervals (CIs) were assessed for a 95% level. Data analysis used a statistical software package (SPSS v20, SPSS Inc., Chicago, IL). Two-tailed *p* < .05 was considered statistically significant.

## Results

### Oakland score in the mild and severe CI group

We first reported the Oakland score and its predictive value among Chinese patients with CI (Table 1). The Oakland score was 8.77 ± 1.63 (mean ± SD) in the mild CI group; however, we observed that the Oakland score was 12.00 ± 3.02 (mean ± SD) in the severe CI group, much higher than that in the mild CI group (*p* < .001) (Table 2).

**Table 1.** Oakland score.

Predictor	Score component value
Age	
<40	0
40–69	1
≥70	2
Gender	
Female	0
Male	1
Previous LGIB admission	
No	0
Yes	1
DRE findings	
No blood	0
Blood	1
Heart rate	
<70	0
70–89	1
90–109	2
≥110	3
Systolic blood pressure	
<90	5
90–119	4
120–129	3
130–159	2
≥160	0
Haemoglobin (g/L)	
<70	22
70–89	17
90–109	13
110–129	8
130–159	4
≥160	0

LGIB: lower gastrointestinal bleeding; DER: digital rectal examination.

**Table 2.** Comparison of clinical and laboratory characteristics in mild and severe CI group.

Characteristics	Mild CI group (*n* = 183)	Severe CI group (*n* = 20)	*p* Value
Gender (male:female)	55:128	6:14	.724
Age (years old)	64.11 ± 10.31	67.20 ± 12.04	.213
Hypertension	78	13	.056
Diabetes	32	9	.132
Coronary heart disease	14	4	.066
Stroke	14	3	.262
Atrial fibrillation	10	3	.099
Past abdominal surgery	27	6	.080
Urgent colonoscopy	74	12	.094
Clinical manifestations			
Abdominal pain	149	18	.343
Diarrhoea	65	12	.187
Fever	59	6	.838
Oakland score	8.77 ± 1.63	12.00 ± 3.02	**<.001**
Haemoglobin (g/L)	142.00 ± 8.14	129.10 ± 11.34	**<.001**
CRP (mg/L)	25.05 ± 32.67	111.83 ± 38.19	**<.001**
Albumin (g/L)	39.58 ± 3.58	35.84 ± 5.88	**<.001**
CAR	0.68 ± 0.97	3.33 ± 1.78	**<.001**

CRP: C-reactive protein; CAR: CRP/albumin ratio. *p* Value was calculated using a *χ*^2^ statistics or Student’s *T* test.

### Clinical characteristics on admission

We compared the clinical features on admission between the mild CI group (*n* = 183) and severe CI group (*n* = 20). The median age was 64.11 ± 10.31 (mean ± standard deviation [SD]) and 67.20 ± 12.04 (mean ± SD) years old, respectively (Table 2). There was no difference of gender between the groups (*p* = .724). There was no substantial statistical difference in hypertension (*p* = .056), diabetes (*p* = .132), coronary heart disease (*p* = .066), stroke (*p* = .262), or atrial fibrillation (*p* = .099) between the mild and severe CI groups (Table 2).

Twenty-seven patients had undergone past abdominal surgeries in the mild CI group, and six patients in the severe CI group had undergone past abdominal surgeries; statistically, the groups did not differ (*p* = .080). In addition, clinical manifestations of fever (body temperature >38.0 °C), abdominal pain, and diarrhoea were documented completely. We found that they did not show a significant difference between the groups (*p* = .838, *p* = .343, *p* = .187) (Table 2).

### Mortality

Two patients died in the severe CI group (10%), one patient from heart failure, the other from acute respiratory distress syndrome (ARDS); both patients were older than 70 years.

### Colonoscopy examination

**Figure 2.**
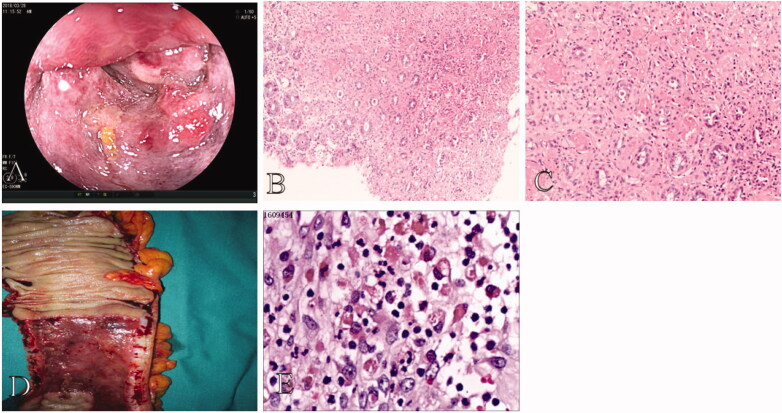
Colonoscopy and pathological findings in a 64-year-old male patient with severe CI. (A) Colonoscopy of severe CI patients showed bluish-black mucosal appearance, mucosal edema, haemorrhage, and lumen prominent stricture with gangrene at the ascending colon. (B,C) Biopsy shows epithelial abscission and haemorrhage in the lamina propria (original magnification: ×100) as well as fibrin thrombi (original magnification: ×200). (D,E) Surgical specimen shows remarkably thickening bowel, bluish-black mucosal changes, and spontaneous bleeding. Specimen shows hemosiderin deposition (original magnification: ×400).

Seventy-four patients and 12 patients received urgent colonoscopy in the mild CI group and the severe CI group, respectively. There was no statistical difference between the groups (*p* = .094) (Table 2). Colonoscopy of severe CI patients showed bluish-black mucosal appearance, prominent mucosal edoema, haemorrhage, and lumen stricture with gangrene at the ascending colon (Figure 2).

### Histopathological findings

**Figure 3.**
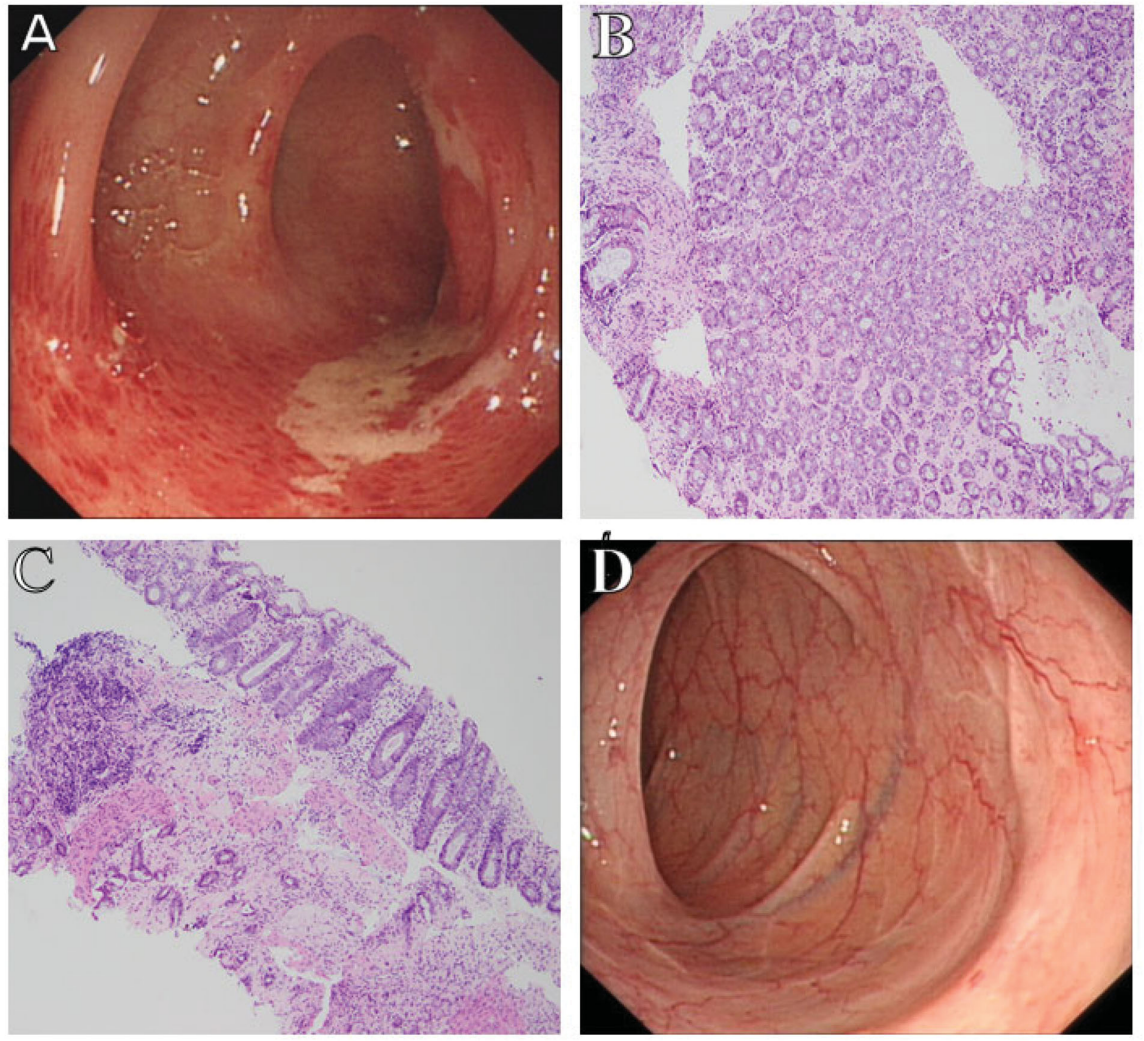
Colonoscopic and pathological findings of a 63-year-old female patient with mild CI. (A) Colonoscopy shows typical longitudinal ulceration (colon single stripe sign), exudate, and congestive edema at the sigmoid colon. (B,C) Biopsy shows epithelial abscission, atrophic glands, and inflammatory cell infiltration in the lamina propria (original magnification: ×200). (D) One-month follow-up of routine colonoscopy shows complete mucosal healing with white scar.

Histopathological findings of the mild CI group were characterized by the following: vessel dilation in 15 patients (8.19%), thickening vessel in seven patients (3.83%), fibrin thrombi in two patients (1.09%), mucosal erosion in 22 patients (12.02%), mucosal ulceration in 23 patients (12.57%), necrosis in 22 patients (11.48%), massive inflammatory cell infiltration in 70 patients (38.25%), and haemorrhage in 23 patients (12.57%) (Table 3). Biopsies from patients with mild CI showed epithelial abscission, atrophic glands, and inflammatory cell infiltration with lamina propria (Figure 3). Pathological findings in the severe CI group were characterized by the following: vessel dilation in two patients (10.00%), a thickening vessel in one patient (5.00%), fibrin thrombi in four patients (20.00%), mucosal erosion in two patients (10.00%), mucosal ulceration in two patients (10.00%), necrosis in three patients (15.00%), massive inflammatory cell infiltration in three patients (15.00%), and haemorrhage in three patients (15.00%) (Table 3). There was significant statistical difference in fibrin thrombi between the two groups (*p* < .001) (Table 3). The odds ratio (OR) of fibrin thrombi was 30.34 (Table 4). However, massive inflammatory cell infiltration occurred more in the mild CI group than in the severe CI group (*p* = .040) (Table 3). Biopsy of one patient with severe CI showed epithelial abscission and haemorrhage in the lamina propria, as well as fibrin thrombi, surgical specimen showed remarkably thickening bowel, bluish-black mucosal appearance, and spontaneous bleeding. Specimen histopathology showed hemosiderin deposition (Figure 2).

**Table 3.** Comparison of pathological findings between mild and severe CI group.

Pathological findings	Mild CI group (*n* = 183)	Severe CI group (*n* = 20)	*p* Value
	*N* (%)	*N* (%)	
Dilated vessel	15 (8.19)	2 (10.00)	.782
Thickening vessel	7 (3.83)	1 (5.00)	.798
Fibrin thrombi	2 (1.09)	4 (20.00)	**<.001**
Mucosal erosion	22 (12.02)	2 (10.00)	.790
Ulceration	23 (12.57)	2 (10.00)	.857
Necrosis	21 (11.48)	3 (15.00)	.643
Inflammation infiltration	70 (38.25)	3 (15.00)	**.040**
Haemorrhage	23 (12.57)	3 (15.00)	.757

*p* Value was calculated using a *χ*^2^ statistics.

**Table 4.** Multivariate logistic regression analysis between mild and severe CI group.

Variables	AUC	OR	95% CI	*p* Value
Oakland score	0.872	1.043	0.801–0.943	**<.001**
CRP (mg/L)	0.950	1.143	0.920–0.980	**<.001**
CAR	0.955	2.830	0.920–0.980	**.017**
Albumin (g/L)	0.719	0.967	0.620–0.820	**<.001**
Fibrin thrombi	0.645	30.34	0.050–0.790	**<.001**

OR: odds ratio; 95% CI: 95% confidence interval.

### Laboratory findings

CAR and CRP were much higher in the severe CI group (3.33 ± 1.78 versus. 0.68 ± 0.97; 111.83 ± 38.19 versus. 25.05 ± 32.67, respectively) (Table 2). However, the AUC of CAR was higher than that of CRP (0.955 versus. 0.950) (Table 4). On the contrary, haemoglobin and albumin were much lower in the severe CI group (129.10 ± 11.34 versus. 142.00 ± 8.14; 35.84 ± 5.88 versus. 39.58 ± 3.58) (Table 2).

### Prognostic scoring model

**Figure 4.**
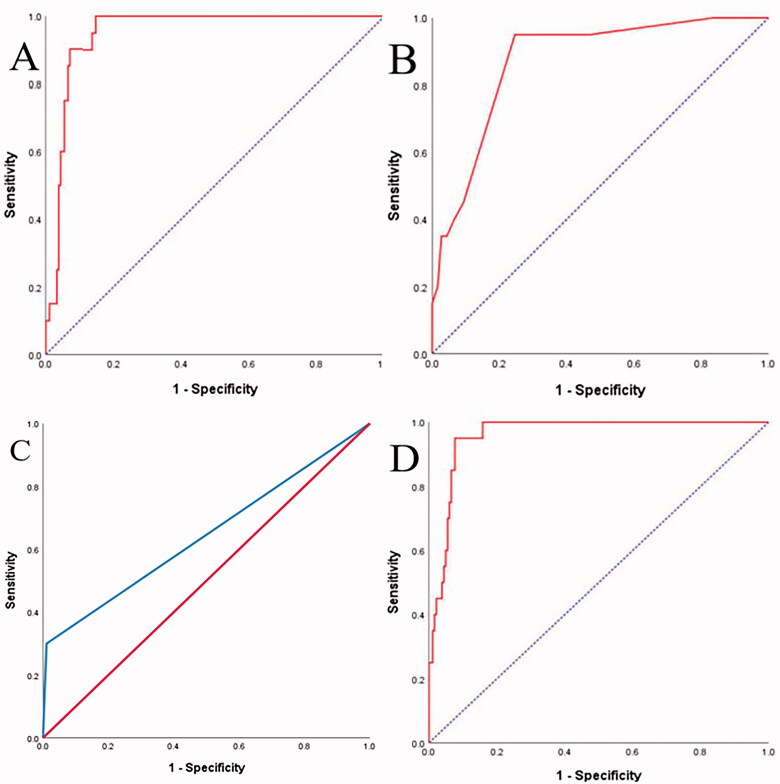
ROC curve of CAR, Oakland score, and the novel predictive scoring model for patients with CI. The AUC of predictive scoring model was 0.960 (95% CI: 0.930–0.990).

We calculated a novel scoring model with three variables based on the estimated logistics model. The preceding variables were Oakland score, CAR, and fibrin thrombi. The final model was 0.042 × Oakland score + 1.040 × CAR + 3.412 × fibrin thrombi; the AUC was 0.960, 95% CIs were 0.930–0.990, the sensitivity was 95%, and the specificity was 92% (Figure 4). Additionally, the threshold value of the above model was 2.74.

## Discussion

CI is the most common manifestation of ischaemic colon disease, and that rate is rising rapidly to approximately three per 1000 hospital admissions in American medical centers [16]. Elderly patients who have multiple underlying diseases are more susceptible to CI [2], and it is generally accepted that many CI patients are over 60 years old [17,18]. Chang et al. reported that cardiovascular diseases and past abdominal surgeries were risk factors for CI; most patients had underlying comorbidities, including hypertension, coronary artery disease, arrhythmia, heart failure, diabetes, cerebral embolism, and history of abdominal surgeries [19]. The mortality in the total population has been reported to be 11.8%, and the mortality among patients with surgical intervention is as high as 37.1% [20].

In this study, we show that mortality in this series of patients with severe CI was only 10.00%. We suggest two possible explanations for the lower mortality in China. First, severe CI cases are not more numerous than those among Western patients because our state is a developing country. Second, the timing of surgical intervention remains controversial; CI is a relatively common surgical entity in the West, it has been reported that intestinal resection for CI was associated high with in-hospital mortality [21]. In addition, we have analyzed the variables of hypertension, diabetes, coronary heart disease, stroke, and atrial fibrillation, and the statistics showed there was no difference on each variable. We found that the medium age in the mild and severe CI groups was 64.11 ± 10.31 and 67.20 ± 12.04 years old, respectively. There was no difference in age in either group (*p*** **=** **.213). Our data were very consistent with previous studies.

It remains controversial whether to perform urgent or standard colonoscopy for acute LGIB patients; European experts recommend that early colonoscopy reduces hospital stay [22]. In Japan, experts have noticed that colonoscopy within 24 h after hospital admission reduced rebleeding compared to patients with acute LGIB who received colonoscopy after 24–96 h [23]. Our results indicated that there was no difference on endoscopy timing regardless of whether urgent or standard colonoscopy was performed on patients with mild or severe CI; our study was remarkably similar to the European study.

Although diagnosis of CI was difficult due to unspecific symptoms and pathological examination, colonoscopy biopsies remained valuable for differentiated diagnosis [24]. American pathologists highlighted that features of acute colitis included epithelial degeneration, epithelial regeneration, edoema, haemorrhage, necrosis, erosion, and neutrophil infiltration [25]. Histopathological findings mostly include superficial necrosis with decreased glands, haemorrhage with lamina propria, fibrin thrombi in small blood vessels, and pseudomembranes [24]. Ulcerative colitis (UC) has a second peak in elderly patients, and CI and UC cannot be easily differentiated. Based only on clinical manifestations, endoscopic features, radiologic findings, it is believed that biopsy is of great value although it is not the gold standard [26]. Some doctors have developed a graded diagnostic classification system for pathological analysis [27]. However, the classification system seems a bit subjective, thus not objective, and it is not yet known whether histopathological findings play an important role in negative outcomes from CI.

However, retrospective studies in the United States demonstrated that patients with CI who had endoscopic biopsies showing necrosis and fibrin thrombi were more likely to have poor outcomes [15]. Unfortunately, there are few previous publications about colonoscopy biopsy and its potential prediction of clinical prognosis in Asia. Our clinical study showed that fibrin thrombi were more likely to signal a negative prognosis in patients with CI (*p* < .001), and neutrophilic inflammation showed a comparatively positive outcome in the severe CI group (*p* = .040). We believe that minimal thrombi combining with local inflammation might cause peritonitis, perforation, sepsis, and shock. In addition, fibrin thrombi might affect potential target-organ failure or even cause death. The need for further large-scale study in the near future is indisputable.

CRP has been widely used to evaluate the severity of inflammation and infection. It was not only a serum marker of clinical deterioration and a factor in decision making for diagnosis or management strategies but also the means for early detection of inflammation and infection in postoperative patients [6]. The CRP normal level (0–10  mg/L) was known to rise due to tissue damage, reaching a peak at 72 h in the postoperative phase and decreasing very soon after. However, CRP detection was not specific, due to intrinsic or extrinsic factors.

Serum albumin was a negative acute-phase protein that was rapidly regulated by inflammatory signals [28]. Low serum albumin was associated with severe inflammation and poor nutrition [12]. Then, CAR became a novel inflammation marker with predictive value in patients with acute pancreatitis and active Crohn’s disease; it was believed to more specific than CRP or albumin alone [29,30]. There are no previous studies of CAR and its predictive value in patients with CI. Our clinical study showed that CAR was 3.33 in the severe CI group, and multivariate logistic regression showed that the AUC of CAR was higher than that of CRP and albumin (0.950, 0.719, respectively); therefore, we presume that CAR detection at admission might be useful to predict CI severity and outcomes. In addition, we need further research into its potential value in large-scale populations.

Acute LGIB was associated with recurrent bleeding and a requirement for blood transfusion [31]. Haemoglobin was used to evaluate bleeding loss, and lower levels of haemoglobin at admission were a strong predictor of severe bleeding [32]. Haemoglobin was much lower in patients with severe CI, based on our study, and the difference was significant (*p* < .001). Therefore, our data were identical to previous study. It is possible to believe that patients with lower haemoglobin count might need blood transfusion or surgery, or even to have life-threatening complications. There was significant difference of haemoglobin between the mild and severe CI group, however, haemoglobin was one part of Oakland score. Therefore, we chose Oakland score for logistic regression analysis.

A high Oakland score was a novel sign of acute LGIB and useful for assessment of whether massive bleeding was present. It was the first score that could be used easily for LGIB. Patients with a score of ≤8 points indicated safe discharge from the emergency department and achievement of a positive outcome. In contrast, patients with a score of >8 points were classified as experiencing massive bleeding, and those patients were more likely to be hospitalized or to have a much higher risk of severe complications or even death [12,13]. A higher Oakland score might remind us that patients need to receive early aggressive management [32]. We found that patients with Oakland scores ≥12 points have a much higher risk for severely adverse outcomes. There is limited data on the Oakland score and its value for predicting severe CI. One assumption we made for the use of Oakland score was that prompt evaluation in the emergency department, and higher points (≥12) were closely associated with adverse outcomes. In addition, we believe that clinicians might easily calculate the Oakland score not only in the emergency centre but also for hospitalized patients.

Our scoring model was based on three independent factors: as Oakland score (points), CAR (serum test), and fibrin thrombi (microscopic finding). The model was 0.042 × Oakland score + 1.040 × CAR + 3.412 × fibrin thrombi. Furthermore, the sensitivity and specificity of this novel scoring model were 95% and 92%, respectively. Our findings were consistent with previous studies, and the aforementioned variables were risk factors for early recognition of patients with severe CI [33,34]. Therefore, future attempts should be directed for prospective study in the near future. In addition, the scoring model for CI outcomes represents a novel tool highly interesting focus for future work and will further enhance our knowledge on CI prognosis. We strongly recommend this scoring model for prediction of CI severity and outcomes.

Our study had some limitations. First, this was a retrospective study with a small sample; we aim to make a prospective study soon. Second, randomized colonoscopy biopsies might contain bias in our study; a nationwide consensus of endoscopic biopsies is necessary, with large-scale research. Finally, interventional technique was not added to the present study. We intend to conduct another clinical trial soon.

We identified laboratory tests (haemoglobin, albumin, and CAR), Oakland score (points ≥12), and histopathological findings (fibrin thrombi) in patients with LGIB caused by colon ischaemia. We did find that combining these novel prediction tools for CI severity and clinical outcomes was valuable. Overall, simple and valuable prognostic tools might remind gastroenterologists to intervene early to improve clinical outcomes and decrease mortality.

## Data Availability

The data support the findings of this study are available (Dr Xinbo Ai having data record), upon reasonable request.

## Abbreviation

CI: colon ischemia; CRP: C-reactive protein; CAR: C-reactive protein to albumin ratio; ROC: Receiver operating characteristics; AUC: area under the curve; SIRS: systemic inflammation response syndrome; ARDS: acute respiratory distress syndrome; CSSS: colon single stripe sign; LGIB: lower gastrointestinal bleeding; DRE: digital rectal examination
